# Preoperative prediction of VETC in hepatocellular carcinoma using non-Gaussian diffusion-weighted imaging at high b values: a pilot study

**DOI:** 10.3389/fonc.2023.1167209

**Published:** 2023-05-25

**Authors:** Chenhui Li, Yan Wen, Jinhuan Xie, Qianjuan Chen, Yiwu Dang, Huiting Zhang, Hu Guo, Liling Long

**Affiliations:** ^1^ Department of Radiology, First Affiliated Hospital of Guangxi Medical University, Nanning, Guangxi, China; ^2^ Department of Pathology, First Affiliated Hospital of Guangxi Medical University, Nanning, Guangxi, China; ^3^ MR Scientific Marketing, Siemens Healthcare Ltd., Wuhan, Hubei, China; ^4^ MR Application, Siemens Healthcare Ltd., Changsha, Hunan, China; ^5^ Key Laboratory of Early Prevention and Treatment for Regional High Frequency Tumor, Gaungxi Medical University, Ministry of Education, Nanning, Guangxi, China; ^6^ Guangxi Key Laboratory of Immunology and Metabolism for Liver Diseases, First Affiliated Hospital of Guangxi Medical University, Nanning, Guangxi, China

**Keywords:** hepatocellular carcinoma, vessels encapsulating tumor clusters, magnetic resonance imaging, diffusion-weighted images, non-Gaussian models, prediction

## Abstract

**Background:**

Vessels encapsulating tumor clusters (VETC) have been considered an important cause of hepatocellular carcinoma (HCC) metastasis.

**Purpose:**

To compare the potential of various diffusion parameters derived from the monoexponential model and four non-Gaussian models (DKI, SEM, FROC, and CTRW) in preoperatively predicting the VETC of HCC.

**Methods:**

86 HCC patients (40 VETC-positive and 46 VETC-negative) were prospectively enrolled. Diffusion-weighted images were acquired using six b-values (range from 0 to 3000 s/mm2). Various diffusion parameters derived from diffusion kurtosis (DK), stretched-exponential (SE), fractional-order calculus (FROC), and continuous-time random walk (CTRW) models, together with the conventional apparent diffusion coefficient (ADC) derived from the monoexponential model were calculated. All parameters were compared between VETC-positive and VETC-negative groups using an independent sample t-test or Mann-Whitney U test, and then the parameters with significant differences between the two groups were combined to establish a predictive model by binary logistic regression. Receiver operating characteristic (ROC) analyses were used to assess diagnostic performance.

**Results:**

Among all studied diffusion parameters, only DKI_K and CTRW_α significantly differed between groups (P=0.002 and 0.004, respectively). For predicting the presence of VETC in HCC patients, the combination of DKI_K and CTRW_α had the larger area under the ROC curve (AUC) than the two parameters individually (AUC=0.747 vs. 0.678 and 0.672, respectively).

**Conclusion:**

DKI_K and CTRW_α outperformed traditional ADC for predicting the VETC of HCC.

## Introduction

Hepatocellular carcinoma (HCC) is the third leading cause of cancer-related death worldwide, and the most frequently diagnosed primary liver malignancy (1). Although partial hepatectomy is the optimal curative strategy, postoperative tumor recurrence and metastasis incidence remains high, resulting in poor prognoses for HCC patients (2, 3). Recently, a novel vascular pattern-based metastasis mechanism, vessels encapsulating tumor clusters (VETC), has been considered an important cause of HCC metastasis (4, 5). However, the HCC patients’ VETC status is not routinely available before operations, which limits its clinical application. Thus, considerable clinical importance is attached to the preoperative VETC assessment *via* a radiology-based approach.

Recent studies have shown that preoperative morphological features of HCC based on magnetic resonance imaging (MRI) or computed tomography (CT) could be used to characterize VETC (6, 7). However, qualitative assessment of morphological features is subjective and limited by differences in interpretation between observers. Diffusion-weighted imaging (DWI), which allows for water molecule diffusion assessment and tissue microstructural complexity characterization, is considered an effective tool to determine tumor histopathological types preoperatively (8, 9). In addition, apparent diffusion coefficient (ADC), which is the most commonly used DWI-derived parameter from a monoexponential model, has been widely used to characterize and classify HCC (10, 11). Nevertheless, Fan et al. (6) found that the conventional ADC value was not an independent predictor of VETC-positive in multivariate analysis although it showed significantly difference between the two groups in univariate analysis, indicating that there are still some limitations in using ADC to differentiate VETC status of HCC. ADC does not reflect water diffusion with non-Gaussian properties in complex sub-cellular microstructures. Therefore, it does not contain all the information on water diffusion (12, 13). To acquire more accurate water diffusion information and map the tissues’ microstructure, several non-Gaussian mathematical models based on high b-values DWI have been established, including diffusion kurtosis imaging (DKI) (14), stretched-exponential model (SEM) (15), fractional order calculus (FROC) model (16), and continuous-time random walk (CTRW) model (17, 18).

Previous studies have shown that these advanced non-Gaussian models are superior to the monoexponential model in detecting the microstructural heterogeneity of various solid tumors, such as glioma, endometrial carcinoma, and hepatocellular carcinoma (18–21). However, to the best of our knowledge, these non-Gaussian models have not been conducted for VETC pattern evaluation. Therefore, we aimed to compare the potential of the monoexponential and non-Gaussian models (DKI, SEM, FROC, and CTRW) in preoperative VETC prediction, as well as to find potential predictors.

## Methods and materials

### Patients

This prospective trial was approved by the ethics committee of The First Affiliated Hospital of Guangxi Medical University (NO. KY-E-245), and informed consent was obtained from all participating individuals. From December 2021 to August 2022, 159 individuals whose CT and/or ultrasonography results indicated primary liver cancer were recruited. All patients received preoperative conventional MRI and 6 b-value DWI in our institute.

Exclusion criteria: (1) the patient received treatment previously (transcatheter arterial chemoembolization or radiofrequency ablation, etc.), 20 cases; (2) the patient was not eligible for surgery or did not receive surgery in our hospital, 25 cases; (3) the interval between the MRI and surgery exceeded 1 month, 5 cases; (4) the HCC lesion was too small (<1 cm), 6 cases; (5) the final pathological results indicated other malignancies instead of HCC, 14 cases; (6) the quality of the MRI image was inadequate for analysis, 3 cases. Ultimately, 86 patients were included in the study.

### Image acquisition

A MAGNETOM Prisma 3T MRI scanner (Siemens Healthcare, Germany) with a body coil (18 channels) and a spine coil (12 channels) was used to examine the patients. A free-breath single-shot echo-planar-imaging (ss-EPI) combined with integrated slice-specific dynamic shimming (iShim) was used to acquire the DWI data for non-Gaussian and monoexponential models simultaneously in three orthogonal directions. The imaging acquisition parameters are as follows: 6 b-values = 0, 200, 600, 1000, 2000, and 3000 s/mm^2^ (with 1, 1, 1, 2, 4, and 6 averages, respectively), repetition time (TR) = 4,900 ms, echo time (TE) = 57 ms, field of view (FOV) = 380 × 261 mm^2^, matrix size (MS) = 128 × 88, slice thickness (ST) = 5 mm, slice gap = 1 mm, parallel imaging acceleration factor = 2, diffusion scheme = monopolar, bandwidth = 2442 Hz/pixel, and scan duration = 4 min and 40s.

For conventional MRI sequences, the information and parameters are listed as follows: The transverse fat-suppressed T2-weighted images were acquired with respiratory-triggered turbo spin-echo sequence (TR = 2,800 ms, TE = 85 ms, FOV = 380 × 380 mm^2^, MS = 320 × 320; ST = 3 mm, and the flip angle (FA) = 120°); the coronal T2-weighted images were acquired with half-Fourier single-short turbo spin-echo (HASTE) sequence (TR = 1,000 ms, TE =95 ms; FOV = 380 × 380 mm^2^, MS = 192 × 192, ST = 5 mm, and FA = 160°); in-phase and out-phase T1-weighted imaging was performed with fast spoiled gradient-recalled dual-echo sequence (TR = 81 ms, TE = 1.3 and 2.5 ms; FOV = 380 × 296 mm^2^, MS = 320 × 224, ST = 3 mm; and FA = 9°); the fat-suppressed axial T1-weighted 3D volume interpolated breath-hold examination (VIBE) sequence (TR = 3.55, TE = 1.30 ms, FOV =380 × 296 mm^2^, MS = 320 × 224, ST = 3 mm, and FA =9°- 30°) was used to capture images at the following phases: pre-contrast, late arterial phase (25 – 35 s), portal venous phase (55 – 65 s), and delayed phase (3 min).

### Image processing and analysis

The data generated from the five diffusion models were analyzed using a post-processing software Body-DiffusionLab (BoDiLab, Chengdu ZhongYing Medical Technology Co., Ltd., Chengdu, China). The corresponding calculation formulas of models are as follows:

(1) Monoexponential model


(1) $$\text{S}(\text{b})={\text{S}}_{0}\exp(-\text{bADC})$$

where S(b) and S0 are the image signal intensities measured with and without diffusion weighting of b value, respectively. ADC is the apparent diffusion coefficient.

(2) Diffusion kurtosis imaging model


(2) $$\text{S}(\text{b})=S_{0}\exp(-\text{bD}+{\text{b}}^{2}{\text{D}}^{2}\text{K}/6)$$

where D represents diffusivity, and K represents kurtosis.

(3) Stretched-exponential model


(3) $$\text{S}(\text{b})={\text{S}}_{0}\exp[{\left(-\text{bDDC}\right)}^{\text{α}}]$$

where DDC represents distributed diffusion coefficient, and α is intravoxel heterogeneity index.

(4) Fractional order calculus model


(4) $$\text{S}(\text{b})={\text{S}}_{0}\exp[-\text{D}{\text{μ}}^{2\;(\text{β}-1)}{\left(\text{γ}{\text{G}}_{\text{d}}\text{δ}\right)}^{2\text{β}}(\text{Δ}-\frac{2\text{β}-1}{2\text{β}+1}\text{δ})]$$

where D represents diffusion coefficient, β represents fractional order derivative in space, and μ is spatial constant. G_d_ is the diffusion gradient amplitude, Δ is the gradient lobe separation, δ is the diffusion gradient pulse width.

(5) Continuous-time random walk model


(5) $$\text{S}(\text{b})={\text{S}}_{0}{\text{E}}_{\text{α}}[-{\left(\text{bD}\right)}^{\text{β}}]$$

where D represents anomalous diffusion coefficient, α and β represent temporal diffusion heterogeneity and spatial diffusion heterogeneity respectively.

The fittings for the four non-Gaussian models were performed using the DWI data across all b-values. For the monoexponential model, fittings were conducted using DWI data with b-values of 0, 600, and 1,000. The parameters included the diffusivity (DKI_D) and kurtosis (DKI_K) from the DKI model, the distributed diffusion coefficient (SEM_DDC) and intravoxel heterogeneity index (SEM_α) from the SE model, the diffusion coefficient (FROC_D), fractional order derivative in space (FROC_β) and spatial constant (FROC_μ) from the FROC model, the anomalous diffusion coefficient (CTRW_D), temporal diffusion heterogeneity (CTRW_α) and spatial diffusion heterogeneity (CTRW_β) from the CTRW model, and the apparent diffusion coefficient (ADC) from the monoexponential model. Two experienced radiologists blinded to the study independently and manually delineated the tumors’ volume of interest (VOI) along the boundary of the whole tumor on each slice of DW images (b-value = 1000 s/mm^2^) using 3D slicer (version 5.0.2). Obvious cystic or necrotic areas were excluded according to the signals from T2- and contrast-enhanced T1-weighted images. Subsequently, the VOIs were applied to all other parametric maps to determine the parametric values, and the mean value was used.

### Clinical and histopathological evaluation

The clinical records of enrolled patients were retrieved from the hospital information system (HIS). Surgically removed hepatic tissues were independently evaluated by 2 experienced pathologists blinded to this study to determine their pathological classification. Any disagreements were discussed in detail, and the data were reviewed again until a consensus was reached. Notably, it is well-documented that the VETC pattern is construed as CD34-positive sinusoid-like vessels forming web-like networks and trapping individual tumor clusters in the whole/part of the tumor (4, 22).

### Statistical analysis

All statistical analyses were generated using SPSS software (Version 23.0, IBM). Categorical and quantitative variables were presented as numbers/percentages and mean values ± standard deviation (SD). The Pearson’s Chi-Square test (including continuity correction when appropriate) was used for categorical data comparisons. On the other hand, the unpaired student’s t-test (for normal distribution data) and Mann-Whitney U test (for nonnormal distribution data) were used for continuous variable comparison between the VETC-positive and VETC-negative groups. A value of P less than 0.05 was considered statistically significant. The intraclass correlation coefficient (ICC) was used to reflect the inter-observer agreement toward the diffusion parameters (poor:< 0.50, moderate: 0.50 - ≤ 0.75, good: 0.75 - ≤ 0.90, and excellent: > 0.90). The diffusion parametric values measured by two radiologists were averaged for further analysis. Binary logistic regression was used to integrate the parameters with significant differences between the two groups for establishing a predictive model. Finally, receiver operating characteristic (ROC) curves were plotted to evaluate the predictive power of every single parameter with a significant difference and their combined model. The maximum Youden index value was used to define the optimal cutoff value, and the related sensitivity and specificity were evaluated. DeLong test was used to assess the predictive power by comparing the area under the ROC curve (AUC).

## Results

### Patient information

In this study, 86 patients were enrolled, including 72 male and 14 female patients (30–77 years old, median age: 52). There were 40 VETC-positive HCC cases (30–77 years old, median age: 52) and 46 VETC-negative HCCs (38–77 years old, median age: 52). The detailed clinical information of the patients is demonstrated in **Table 1**. We did not observe a statistical difference in the clinical characteristics between the VETC-positive and VETC-negative groups.

**Table 1:** Patients’ clinical characteristics.

Characteristics	VETC-positive (n=40)	VETC-negative (n=46)	P Value
Mean age (years) *	53 ± 9	52 ± 9	0.645
Gender			0.775
Men	33 (82.5)	39 (84.8)	
Women	7 (17.5)	7 (15.2)	
HBV or/and HCV infection			0.919
Yes	36 (90.0)	40 (87.0)	
No	4 (10.0)	6 (13.0)	
Cirrhosis			0.147
Yes	38 (95.0)	38 (82.6)	
No	2 (5.0)	8 (17.4)	
No. of lesions			0.567
1	34 (85.0)	41 (89.1)	
≥ 2	6 (15.0)	5 (10.9)	
Serum AFP			0.582
>100 ng/mL	22 (55.0)	28 (60.9)	
≤100 ng/mL	18 (45.0)	18 (39.1)	
BCLC stage			0.476
0 or A	24 (60.0)	31 (67.4)	
B or C	16 (40.0)	15 (32.6)	

Excepted where indicated, data are numbers of patients with percentages in parentheses. *Data are means ± deviation. The age is compared by using independent sample t test. HBV, hepatitis B virus; HCV, hepatitis C virus; AFP, α-fetoprotein; BCLC, Barcelona Clinic Liver Cancer; VETC, vessels encapsulating tumor clusters.

### DWI parameters in predicting VETC

**Figure 1:**
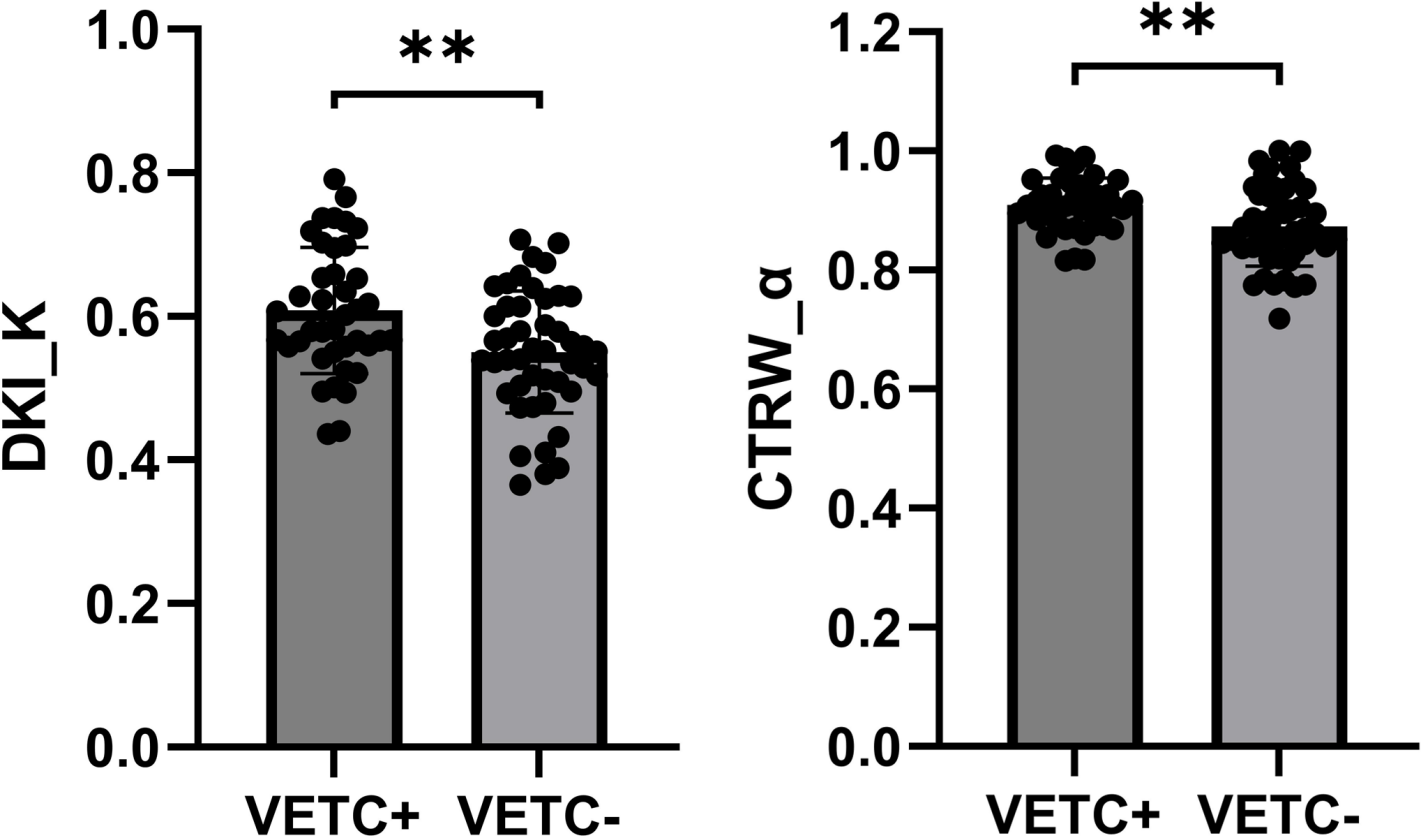
Box plot showing the DKI_K and CTRW_α values of VETC-positive and VETC-negative groups. **statistically significant difference (P<0.005).

**Figure 2:**
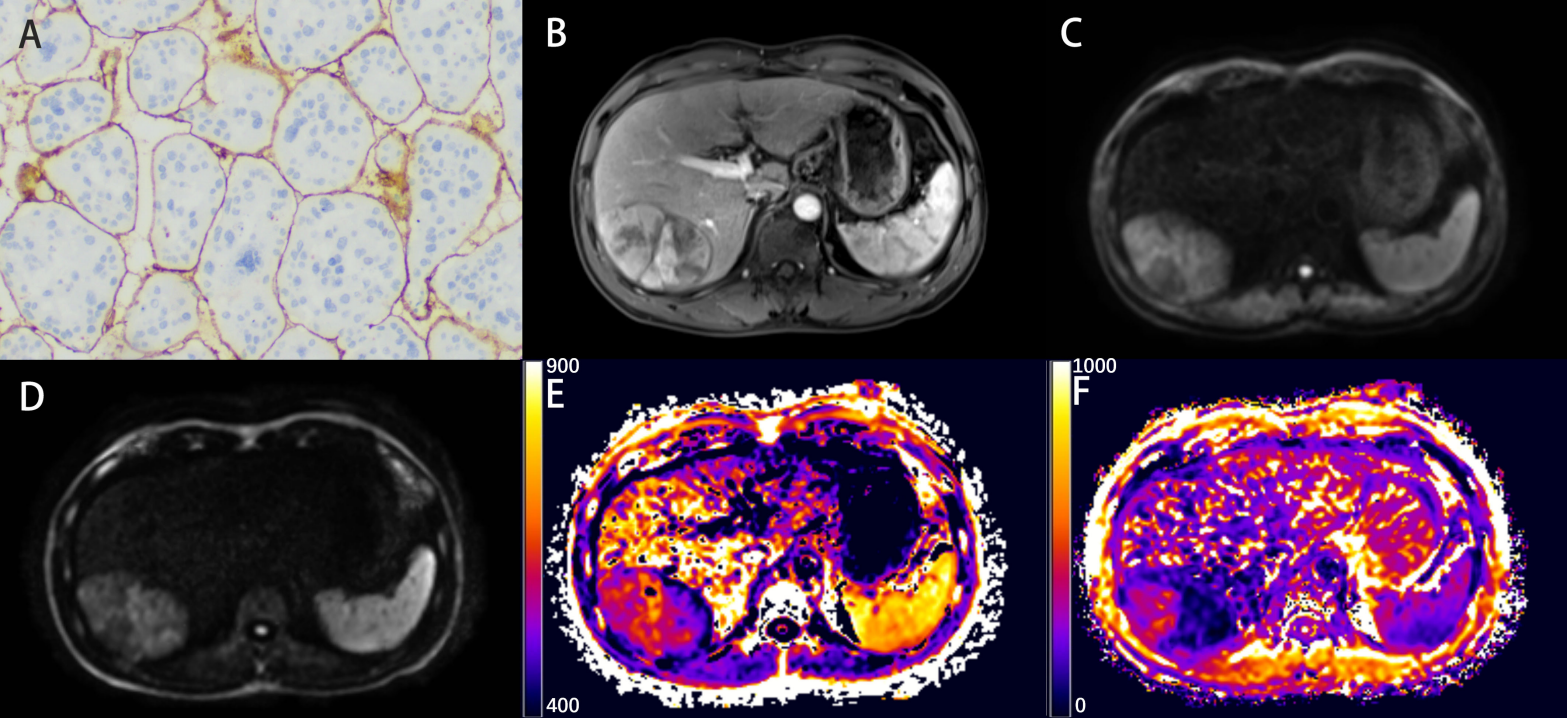
Representative histopathological and radiological images of an HCC case with VETC (51-year-old male). **(A)** Representative immunohistochemical staining of CD34 (original magnification × 100) of the resected hepatic tissue showing typical VETC pattern tumor cluster captured by a web-like vascular network. **(B)** The arterial phase showing inhomogeneous and marked enhancement of the lesion in the right lobe of the liver. **(C, D)** The DW image obtained with b-values of 1000 s/mm^2^ and 3000 s/mm^2^, respectively. **(E, F)** The DKI_K map **(E)**, and CTRW_α map **(F)**.

**Figure 3:**
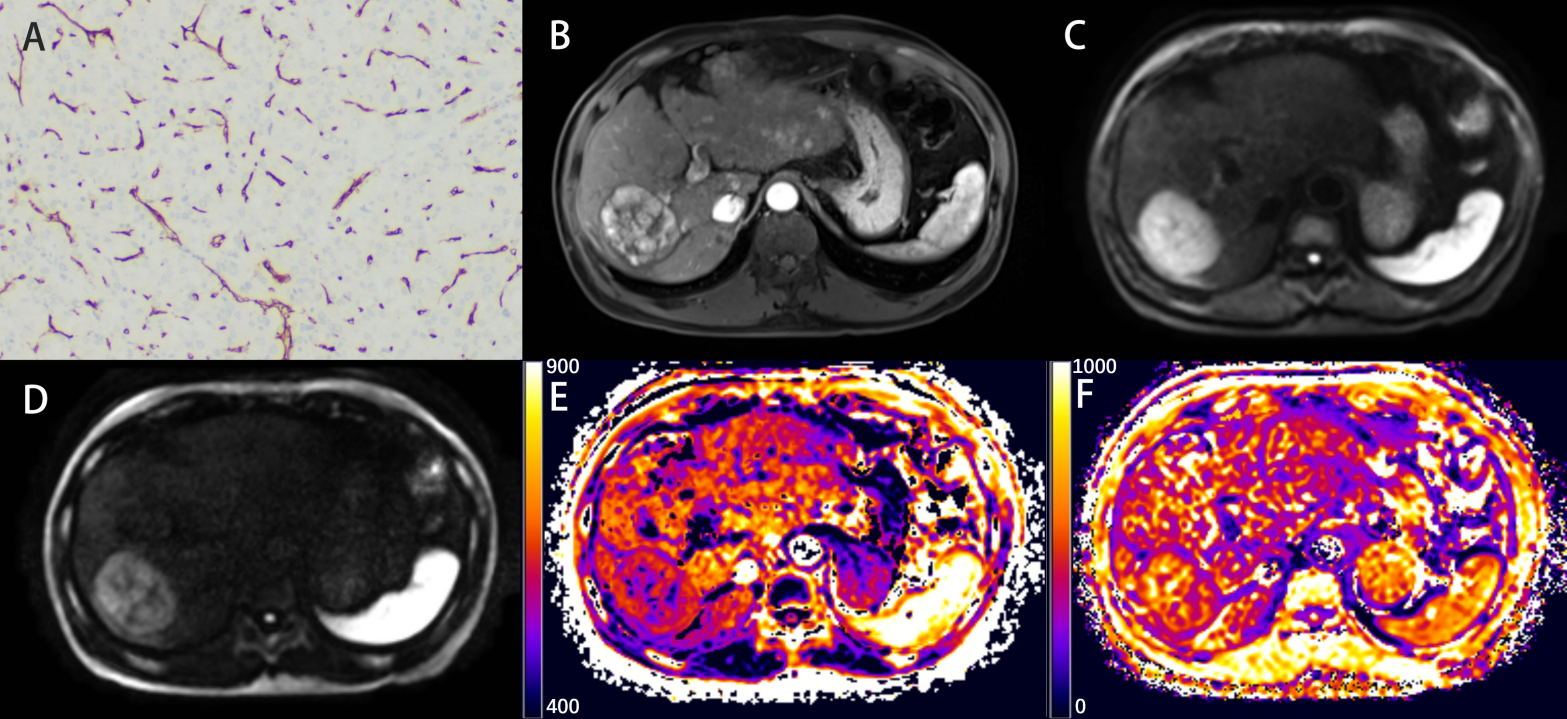
Representative histopathological and radiological images of an HCC case without VETC (60-year-old male). **(A)** Representative immunohistochemical staining of CD34 (original magnification × 100) of the resected hepatic tissue showing a classical capillary vascular pattern with discrete lumens. **(B)** The arterial phase indicating inhomogeneous and marked enhancement of the lesion in the right lobe of the liver. **(C, D)** The DW image obtained with b-values of 1000 s/mm^2^ and 3000 s/mm^2^, respectively. **(E, F)** The DKI_K map **(E)**, and CTRW_α map **(F)**.

Detailed parameters derived from each non-Gaussian model and ADC values measured by two observers were demonstrated in **Table 2**. Significant differences in the mean values of DKI_K and CTRW_α were observed between HCCs with or without the presence of VETC (**Figure 1**). Moreover, inter-observer agreements between the two observers were excellent (DKI_K, ICC: 0.948; 95% CI: 0.921–0.966; CTRW_α, ICC: 0.904; 95% CI: 0.853–0.937) (**Table 2**). Furthermore, the mean valuse of ADC, DKI_D, CTRW_D, CTRW_β, and all other parameters derived from SEM and FROC model did not show significant intergroup differences (**Table 3**). **Figures 2**, **3** display representative MRI scanning results of HCCs acquired from a VETC-positive patient (51-year-old male) and a VETC-negative patient (60-years-old male), respectively.

**Table 2:** Diffusion parameters between the VETC-positive and VETC-negative groups and agreements between two radiologist.

Parameters	Radiologist 1			Radiologist 2			ICC
	VETC-negative	VETC-positive	P value	VETC-negative	VETC-positive	P value	
ADC (μm^2^/ms)	1.04 ± 0.21	1.01 ± 0.19	0.489	1.05 ± 0.20	1.05 ± 0.20	0.836	0.817
DKI_D (μm^2^/ms)*	1.48 ± 0.43	1.46 ± 0.34	0.986	1.50 ± 0.39	1.52 ± 0.33	0.795	0.784
DKI_K	0.55 ± 0.09	0.62 ± 0.09	0.001	0.55 ± 0.09	0.60 ± 0.09	0.008	0.948
SEM_DDC (μm^2^/ms)	1.64 ± 0.55	1.48 ± 0.51	0.164	1.66 ± 0.60	1.55 ± 0.48	0.367	0.859
SEM_α	0.55 ± 0.14	0.58 ± 0.13	0.248	0.54 ± 0.14	0.58 ± 0.12	0.188	0.964
FROC_D (μm^2^/ms)	1.00 ± 0.28	0.97 ± 0.22	0.486	1.02 ± 0.25	1.00 ± 0.22	0.794	0.727
FROC_β*	0.67 ± 0.12	0.66 ± 0.10	0.411	0.66 ± 0.13	0.65 ± 0.10	0.550	0.922
FROC_μ (μm)	3.47 ± 0.54	3.66 ± 0.41	0.068	3.50 ± 0.57	3.67 ± 0.31	0.075	0.929
CTRW_D (μm^2^/ms)*	1.31 ± 0.34	1.25 ± 0.27	0.494	1.32 ± 0.29	1.30 ± 0.29	0.616	0.793
CTRW_α	0.87 ± 0.07	0.91 ± 0.05	0.006	0.88 ± 0.07	0.91 ± 0.04	0.007	0.904
CTRW_β	0.66 ± 0.14	0.64 ± 0.13	0.615	0.64 ± 0.14	0.63 ± 0.12	0.786	0.917

Excepted where indicated, data are compared by using were compared using an independent sample t-test. *Date were compared by using a Mann-Whitney U test. ADC, apparent diffusion coefficient; DKI_D, diffusivity; DKI_K, kurtosis; SEM_DDC, distributed diffusion coefficient; SEM_α, intravoxel diffusion heterogeneity index; FROC_D, diffusion coefficient; FROC_β, fractional order derivative in space; FROC_μ, spatial constant; CTRW_D, anomalous diffusion coefficient; CTRW_α, temporal diffusion heterogeneity index; CTRW_β, spatial diffusion heterogeneity index; ICC, intraclass correlation coefficient.

**Table 3:** Mean values of diffusion parameters between the VETC-positive and VETC-negative groups.

	VETC-negative	VETC-positive	P value
ADC (μm^2^/ms)	1.05 ± 0.18	1.03 ± 0.19	0.623
DKI_D (μm^2^/ms)*	1.49 ± 0.35	1.49 ± 0.33	0.986
DKI_K	0.55 ± 0.08	0.61 ± 0.09	0.002
SEM_DDC (μm^2^/ms)	1.65 ± 0.52	1.51 ± 0.48	0.221
SEM_α	0.55 ± 0.14	0.58 ± 0.12	0.209
FROC_D (μm^2^/ms)	1.01 ± 0.22	0.99 ± 0.21	0.583
FROC_β*	0.67 ± 0.12	0.66 ± 0.10	0.499
FROC_μ (μm)	3.48 ± 0.54	3.66 ± 0.34	0.061
CTRW_D (μm^2^/ms)*	1.31 ± 0.27	1.27 ± 0.27	0.431
CTRW_α	0.87 ± 0.07	0.91 ± 0.04	0.004
CTRW_β	0.65 ± 0.13	0.64 ± 0.12	0.686

Excepted where indicated, data are compared by using an independent sample t-test. *Date were compared using a Mann-Whitney U test.

### ROC analysis

**Figure 4:**
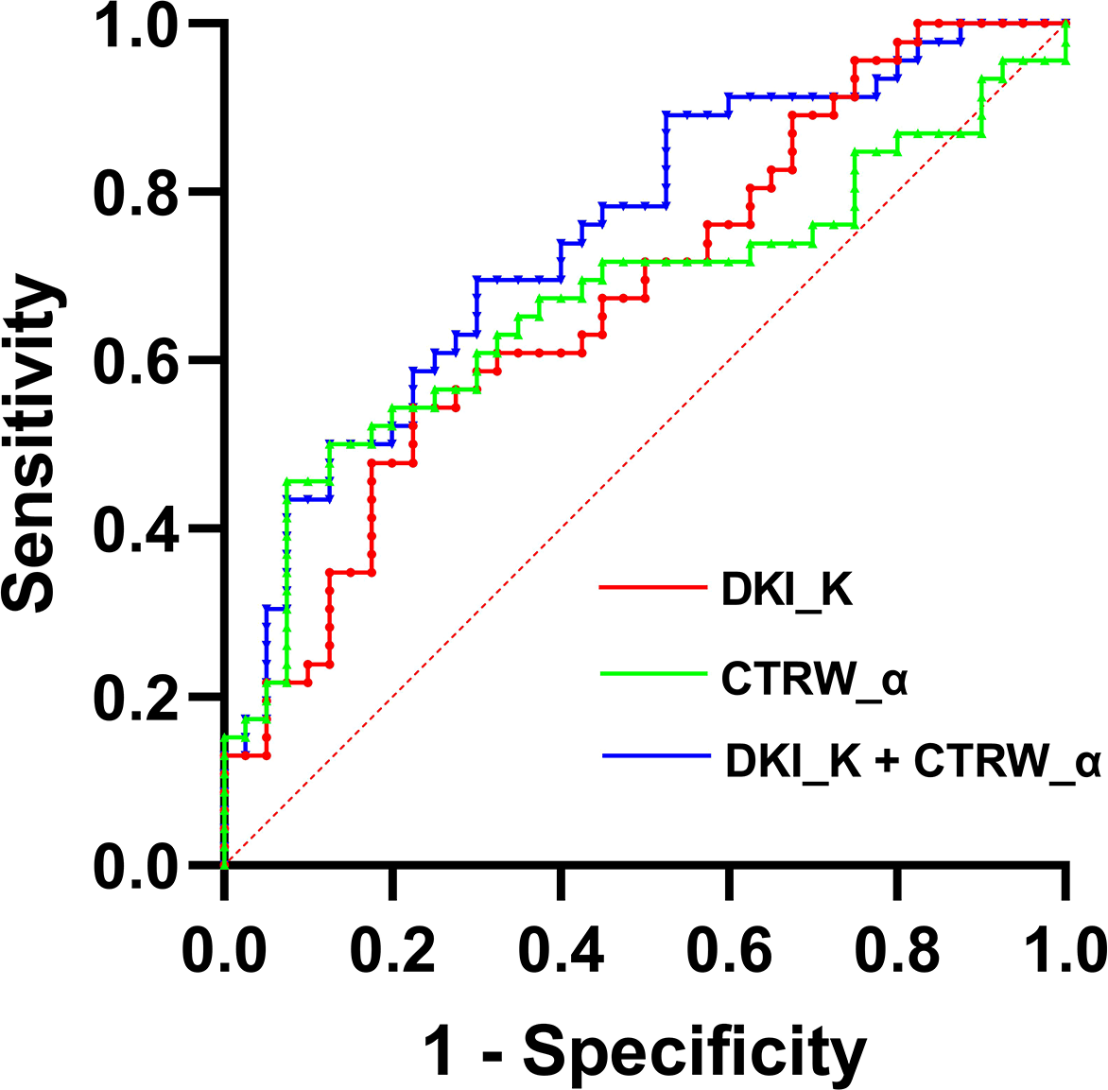
The ROC curves for DKI_D, CTRW_α, and their combination for differentiating VETC-positive and VETC-negative HCCs. AUCs for the corresponding ROC curves are 0.747 (DKI_K+CTRW_α), 0.678 (DKI_K), and 0.672 (CTRW_α).

We also evaluated the performance of the diffusion parameters with significant intergroup differences in determining VETC in HCC cases by comparing their ROC curves (**Figure 4**). As shown in **Table 4**, AUC, sensitivity, specificity, optimal cutoff value, and Youden index were analyzed. We found that compared with the AUC of individual DKI_K and CTRW_α (0.678 and 0.672), combining DKI_K and CTRW_α resulted in a larger AUC (0.747). Delong test showed that the AUCs between DKI_K, CTRW_α and combining DKI_K and CTRW_α had no significant difference (all P>0.05).

**Table 4:** Diagnostic performance for differentiating VETC-positive and VETC-negative HCCs.

	AUC (95% CI)	Accuracy	Sensitivity	Specificity	Youden index	Optimal cutoff value
DKI_K	0.678 (0.566-0.791)	65.1%	77.5%	54.3%	0.318	0.557
CTRW_α	0.672 (0.556-0.787)	67.5%	92.5%	45.7%	0.382	0.854
DKI_K + CTRW_α	0.747 (0.644-0.850)	69.8%	70.0%	69.6%	0.396	0.501

## Discussion

In this study, we compared the prediction capacity of parameters derived from monoexponential model and four non-Gaussian diffusion models in VETC presence. The results showed that among all parameters, only DKI_K and CTRW_α were potential predictors of VETC positivity. Besides, the combination of these two parameters had moderate diagnostic ability for VETC (AUC=0.747).

One previous study reported by Fan et al. (6) showed that the VETC-positive cases had significantly lower ADC values than VETC-negative cases. Consistent with this finding, we also observed lower ADC values in the VETC-positive group compared with the VETC-negative group. However, the difference failed to reach statistical significance, possibly due to our limited sample size and the differences in the factors employed in the study, such as ROI selection, b-values, and scanning protocol. Furthermore, it is well-documented that water molecules in tumor tissues with complex structural heterogeneity exhibit a non-Gaussian phenomenon (23). Therefore, using a monoexponential DWI model based on Gaussian assumptions to distinguish these microstructural variations might lead to controversial insights (24–28). In addition, compared with ADC values, the non-Gaussian diffusion model-derived parameters, especially at higher b-values, can better illustrate the tumors’ microstructural complexity and provide a stronger pathological correlation (8, 29, 30). Consistent with this speculation, our study found that the VETC prediction capabilities of DKI_K and CTRW_α values were better than conventional ADC values. An increasing body of evidence has implicated that VETC is a heterogeneous pattern of angiogenesis and might be associated with increased heterogeneity and aggressiveness in HCCs (22, 31). By introducing a unique parameter, kurtosis (DKI_K), the DKI model establishes the relationship between the degree of deviation from the Gaussian distribution of the water molecule displacement and microstructural heterogeneity in tumor tissues (14, 23). Increased DKI_K is considered to occur in more heterogeneous environments with multiple or large interfaces (32). In VETC-positive HCCs, the vascular endothelial cells form a web-like complex network (22), leading to higher DKI_K values. Accumulating evidence revealed that DKI_K possesses higher diagnostic potential than ADC and DKI_D in predicting variations in tissue microstructure of HCC (33, 34), which echoes our findings. Moreover, based on the CTRW theory (17), CTRW_α describes the probability of water molecules being retained or released while they diffuse through tissue structures, and CTRW_β reflects the heterogeneity of diffusion “jump” length in each move. Therefore, CTRW model can reflect the heterogeneity of the intra-voxel diffusion in both time and space. One study by Karaman et al. (18) showed that the CTRW-derived parameters provided higher AUC than ADC in distinguishing brain tumor heterogeneity, which was similar to our results.

In our results, expect DKI_K and CTRW_α, the other non-Gaussian model parameters did not correlate with the VETC of HCC. On the one hand, the failure to achieve statistical significance in other parameters may be partially caused by the limited sample size. On the other hand, each model’s parameters focus on different aspects of non-Gaussian properties and are not necessarily different between VETC subgroups defined by the tumor vascular morphology. So far, the non-Gaussian diffusion models have rarely been substantially studied in HCC, and the biological interpretation of their various parameters remains intriguing. Therefore, the potential underlying mechanisms and the association between the parameters above and VETC requires further investigation.

There is an increasing need for radiologists to be able to provide additional information to oncologists which could be used to evaluate tumor grading, classifications, and prognoses. Unfortunately, conventional MRI based on morphological features alone cannot satisfy this challenge due to the limitation of achievable voxel size. In contrast, high b-value DWI demonstrated promising capability in distinguishing tumor heterogeneity and predicting tumor aggressiveness (8). However, high b-value DWI requires the appropriate selection of b-values, acceptable acquisition time, and adequate signal-to-noise ratio (SNR) (8), and its operation usually takes a long time under stringent conditions. Thus, under these scenarios, its application is not prioritized in clinical management. A novel aspect of our study is using a scanning protocol that acquires all b-values required for the four non-Gaussian diffusion models within a clinically acceptable acquisition time. According to previous studies (8, 35, 36), the 6 b-values can be divided into low (0–200 s/mm^2^), moderate (600–1000 s/mm^2^), and high (2000–3000 s/mm^2^) b-value clusters. Our study used a prototype ss-EPI sequence with ishim (37, 38) and motion correction algorithm to reduce image distortion and increase SNR. This scheme achieved sufficient imaging quality and adequate inter-observer agreement, indicating the feasibility of the conventional application of advanced diffusion models in liver imaging. Additionally, five diffusion models were calculated from data obtained in a single acquisition, and the diagnostic capacities of various parameters were compared to predict the incidence of VETC in this study. This comprehensive comparison is more informative and representative than an individual survey. Furthermore, combining multiple diffusion model-derived parameters was shown to capture different properties, such as cellularity, vascularity, microstructures, and heterogeneity, all of which could lead to higher prediction accuracy in malignancy diagnoses (20, 39).

Our study has several limitations. First, the sample size was limited. And to determine VETC more accurately, only patients who underwent tumor resections in our hospital were enrolled, and cases with needle biopsies were excluded, which may introduce some sampling bias. In the future, we will expand the sample size to improve the reliability of the results. Second, diffusion-weighted imaging data were obtained under free-breathing, which could cause some interference in the model’s curve fitting. Although a consensus was not reached, some investigators recommended the free-breathing scheme due to its reproducibility and shorter acquisition duration (40, 41). Last, only the mean value in VOI region was assessed, which may reduce the diagnostic efficiency of the parameters because mean value may cannot reflect the heterogeneity of whole tumor. In future studies, we plan to introduce more advanced analytical methods, such as histogram analysis, and habitat analysis, to evaluate the diagnostic efficiency of these diffusion models more comprehensively.

## Conclusion

In conclusion, this pilot study showed that DKI_K and CTRW_α values derived from non-Gaussian diffusion models are superior to the traditional ADC value in predicting the VETC of HCC.

## Data availability statement

The raw data supporting the conclusions of this article will be made available by the authors, without undue reservation.

## Ethics statement

The studies involving human participants were reviewed and approved by First Affiliated Hospital of Guangxi Medical University Ethics Review Committee. The patients/participants provided their written informed consent to participate in this study.

## Author contributions

CL and YW contributed equally to this work. Study concept and design, CL, YW, and LL. Drafting of the manuscript, CL. Critical revision of the manuscript, all authors. Image obtaining and postprocessing, CL, YW, JX, and QC. Statistical analysis, HZ. Administrative, technical, or material support, LL, HZ, HG, and YD. All authors contributed to the article and approved the submitted version.
